# Organisation of services for people with cardiovascular disorders in primary care: transfer to primary care or to specialist-generalist multidisciplinary teams?

**DOI:** 10.1186/1471-2296-15-158

**Published:** 2014-09-22

**Authors:** Egle Price, Richard Baker, Jane Krause, Christine Keen

**Affiliations:** Department of Health Sciences, University of Leicester, 22-28 Princess Road West, LE1 6TP Leicester, UK; 6 Northage Close, Quorn, Loughborough, Leicestershire, LE128AT UK; NIHR RDS East Midlands, Faculty of Health & Life Sciences, Innovation Centre, De Montfort University, The Gateway, Leicester, LE1 9BH UK

**Keywords:** Primary care, Cardiology, Chronic disease care, Community, Multidisciplinary teams

## Abstract

**Background:**

An ageing population and high levels of multimorbidity increase rates of GP and specialist consultations. Constraints on health care funding are leading to additional pressure for the adoption of safe and cost-effective alternatives to specialist care, in some cases by shifting services to primary care.

**Discussion:**

In this paper we argue, having searched for evidence on approaches to shifting care for some people with cardiovascular problems from secondary to primary care, that a collaborative, multidisciplinary approach is required to achieve high quality outcomes from cardiovascular care in the primary care setting. Simply transferring patients from specialist care to management by primary care teams is likely to lead to worse outcomes than services that involve both specialists and primary care teams together, in planned and effectively managed systems of care.

The care of patients with certain chronic conditions in the community, if appropriately organised, can achieve the same health outcomes as ambulatory care by hospital specialists. However, shared care by GPs and specialists for patients with chronic heart failure after discharge from hospital can deliver better patient survival. The existing models of shared care include specialists working in an ambulatory care setting (in Central and Eastern Europe) or in hospital based outreach clinics, and cardiology care organised by GPs in the UK and Australia, which have demonstrated reductions in referral rates.

**Summary:**

Current research supports the idea of the management of certain chronic health conditions in primary care based on the integration of GPs and specialists into multidisciplinary teams, based on availability of reliable evidence about cost-effectiveness, health care outcomes, patient preference and incentives for GPs. Evaluation of such schemes is mandatory, however, to ensure that the expected benefits do materialise.

## Background

In many countries, increasing demand for the provision of high quality health care services combined with constraints on health care funding are driving health care systems to seek suitable alternatives to high cost specialist care. Health care systems with better developed primary care tend to have lower health care costs [1], and stronger primary health care is associated with lower all-cause mortality, as well as cause-specific premature mortality, including cardiovascular diseases [2, 3].

In economically developed countries, cardiovascular disorders account for substantial proportions of health care expenditure [4–6]. Higher costs for secondary care services in comparison with the management of problems in primary care [3], difficulties in accessing specialist services, including long waiting times for specialist consultations [7], fragmented care due to lack of communication across the health care system [8] as well as a small number of conditions that do not have clearly defined pathways or responsible health care providers, strongly support the shift of services from secondary to primary care [3].

In England, the increasing use of primary care is evident from the rise in the total number of GP consultations per person per year - from 3.9 in 1995 to 5.4 in 2007 [9]. The ageing population, combined with the longevity of many people who develop chronic health conditions that require specialised care, also result in an increasing need for specialist consultations, total outpatient appointments rising from around 55 million in 2007/8 to 90 million in 2011/12 [10].

In consequence, due to increasing demand for consultations and investigations, health care systems are struggling to provide required services within reasonable time limits [7]. Expanding secondary care services in proportion to increasing demand is not the ideal solution, as increasing the capacity and availability of hospital appointments, although tending to increase costs, does not automatically lead to improved access for all patient groups. Paradoxically, increased access to health care services has been shown to stimulate increased demand [11–14]. Major re-structuring of primary care services is being considered, or is underway, in several countries, partly in order to increase capacity for managing chronic conditions. For example, federations or other forms of practice networking are emerging in England, with similar developments in New Zealand and Canada [15]. The patient centred medical home movement is extending in the United States [16].

But is the direct transfer of patients with complex chronic conditions from secondary to primary care a sensible policy? In this paper we argue, having searched for evidence on approaches to shifting care for some people with cardiovascular problems from secondary to primary care, that a collaborative, multidisciplinary approach is required to achieve high quality outcomes from cardiovascular care in the community. To identify relevant evidence, we referred to articles we had already identified and drew on a search of the following bibliographic databases for papers reporting studies of any design that addressed our question: Medline, Social care online, Current controlled trials metaregister, ASSIA (Proquest), Cochrane, HMIC, Biomed central, Google, Europe PMC, HTA, NIHR portfolio database. The searched included articles published 1994–2014. Keywords used for the search were: 'heart failure', 'heart', 'cardiac', cardiovascular', 'chronic cardiovascular conditions', 'multidisciplinary', 'collaborative', 'models of care', 'service'.

## Discussion

### Is the shift of management of certain chronic conditions to primary care feasible?

During the last decade a shift of some services from secondary to primary care has tended to occur, the rationale being to make better use of health care resources [17]. In England, for example, the GP led clinical commissioning groups introduced in 2013 are charged with maximising the efficiency of services, and in consequence are considering new ways of structuring care. The necessity of finding appropriate safe ways for delivering specialised ambulatory services outside the hospital setting has become widely accepted, illustrated by the Clinical Commissioning Guidelines from the British Cardiovascular Society: "it is likely that some patients currently seen in secondary care cardiology clinics could be managed in appropriately staffed community based services or discharged from follow up altogether” [18].

Evidence suggests that some services provided by GPs are more cost effective [3]. General practitioner led hospitals in Norway provided health care at lower cost compared to alternative modes of care, due to avoidance of some hospital costs [19]. Care delivered by GPs instead of hospital specialists in hospital-based emergency departments, was shown to be more cost effective due to lower rates of investigation, lower referral rates to secondary services, and no significant difference in health outcomes or patient satisfaction [20, 21]. Shifting care across specialist-general practice and secondary-primary care boundaries is also possible and has been shown to be cost effective without an adverse effect on outcome [3]. This was shown to be true for management of chronic health care problems including asthma and diabetes in studies conducted in the UK [22–24] and other countries [25].

### The existing evidence about chronic disease management in community setting

A well-known example of an approach to the management of chronic health conditions outside the specialist setting is Wagner's Chronic Care module (CCM) [26], which has been applied to, among other conditions, congestive heart failure, asthma and diabetes. Developed more than a decade ago, the CCM is a widely adopted approach to improving ambulatory care that has guided clinical quality initiatives in the United States and around the world. The accumulated evidence of the CCM’s effectiveness in articles published since 2000 appears to support the CCM as an integrated framework to guide practice redesign [27]. Although work remains to be done in areas such as cost-effectiveness, these studies suggest that redesigning care using the CCM leads to improved patient care and better health outcomes.

At the same time, the scientific evidence about the consequences of transferring specialised care into primary care is limited in amount as well as limited to circumscribed areas, for example, minor surgery or the management of a few particular chronic diseases [28], rather than extensive studies of more complex health conditions or complete patient populations. In the case of episodic minor surgery, the results are quite conclusive - shorter waiting times, fewer resources consumed, less expense for patients and higher patient satisfaction rates [29]. However, the example of diabetes management in primary care, which has proved to be as effective in terms of clinical and psychological aspects as hospital-based care [23, 30] and reducing costs of health care [31], raises the presumption that at least some other common chronic conditions could be managed equally successfully in primary care as well, provided any transfer of care is associated with thorough evaluation, including analysis of cost-effectiveness.

### Chronic cardiovascular conditions

The burden of cardiovascular diseases for society is growing. They were estimated to cost the EU €169 billion annually (data 2003), with healthcare accounting for 62% of costs [32]. Chronic heart failure in particular is now recognized as a major public health problem [33]. The annual direct cost of heart failure to the NHS in 2000 has been estimated at about 1.9% of total NHS expenditure [33, 34].

Studies of the management of chronic cardiovascular conditions outside hospitals provide limited evidence about shared care. The majority of current publications have been based on simple comparisons of health care outcomes for patients followed in hospital outpatient services versus primary care. As a result of this approach, they support the view that management of chronic cardiovascular conditions such as heart failure and some other cardiovascular diseases are better if performed by specialists than GPs [8, 35–38]. These studies focus exclusively on specialist versus GP care, but what has primary care to offer for *shared* care (described as the joint participation of primary care physicians and specialty care physicians in the planned delivery of care)?

Systematic reviews of research studies focusing on shared care for specific health problems do not show consistent evidence of improvement in health outcomes (both physical and mental) or psychosocial domains, but do demonstrate improvement in prescribing and patient adherence [39]. However, the same resource states that shared care for certain patient groups, including the elderly and people with moderate to severe congestive heart failure was shown to be more effective than care by primary health care physicians or specialty physicians alone.

Other studies support efficiency and improvement in health outcomes in provision of outpatient care across boundaries [40]. A study performed in Veteran hospitals in the US analysed survival of almost 12 thousand patients with chronic heart failure after discharge from hospitals. Patients were divided into four groups based on whether after discharge they were followed up in the community by cardiologists alone, GPs alone, mixed cardiology and GP care, or none. The unadjusted mortality rate was highest for patients in the group with no GP/cardiologist follow up (29%) and lowest for patients in the mixed (both GP and cardiologist follow up) group (19%). Therefore, although the second lowest mortality rate was for the patient group followed up by cardiologists alone, shared care achieved the best patient survival. The pattern of outpatient care was concluded, therefore, to be important for the survival of patients with heart failure.

### Models of cardiovascular disease management in community - do we have to reinvent the wheel?

Current internationally existing models of organisation of ambulatory care for patients with chronic cardiovascular conditions in primary care seem to have three different approaches.

One of the dominant models of care of the disease management programs for patients with chronic cardiovascular conditions involve hospital based clinics and outreach services (comprising home visits and telephone contacts) led by nurses and clinical pharmacists with cardiologist oversight [41]. In this model of care, the GP has assumed a secondary and often disconnected role in managing co-morbidities and acute illnesses unrelated to either secondary prevention or palliation of chronic heart failure [42].

Another, intermediate approach, is the long existing Soviet model still present in different forms in Central and Eastern Europe, that brings primary and specialist services together in a single location [43]. Whether called Health Centres, Diagnostic centres, Outpatient clinics or Polyclinics, they provide specialist care outside the hospital setting and are expected to bridge the gap between primary and specialised care. Most systems retain the gate-keeper role of GPs to regulate access to specialists [44]. Easy accessibility for patients, proximity of specialists in urgent cases or for professional advice for GPs, together with the possibility of immediate personal feedback from specialists to primary care teams seem to be the main advantages of the arrangement.

The third approach may be considered almost the opposite strategy to both presented above; instead of relying on care provided by *specialists* in community setting [45, 46], the *services* are shifted to the primary care level, making primary care physicians (GPs) responsible for organising the service provision by a multidisciplinary team [47, 48].

There is an increasing number of publications across the world providing the evidence to indicate the efficiency of management of chronic heart failure in primary care. A recent study in Sweden demonstrated that management of chronic heart failure in primary care was found to have beneficial effects in terms of reducing the number of healthcare contacts and hospital admissions, and improving cardiac function in patients with systolic heart failure [49].

Examples of a primary care based collaborative model already exist in the UK, one of which is Kent Community Cardiology Service. This is based on three general practitioners with special interest (GPwsi) in cardiology and was established in 2006, serving about 220 000 patients [50]. A reduction of cardiology referral rates and high patient satisfaction were reported as a result of services provided for number of cardiovascular conditions. These findings are in line with experience of other GPs with special interest [51] as well as studies in other countries investigating the outcomes of GP-specialist collaboration for cardiovascular disease management [52].

### Multidisciplinary teams - solution to the problem?

The multidisciplinary team is a widely used concept that implies the collaboration of different health care professionals, and depending on the task, they can be led by a specialist, GP, nurse or any other team member. The participation of medical specialists in consultative and educational roles outside conventional referrals is an essential element contributing to better outcomes. This approach is supported by the British Cardiovascular Society [53]. Nurses are key members of the teams, and community-based nurse led models of care bring significant benefits to the services [54, 55] and supplement the care provided by physicians and other health care professionals [56].

Multidisciplinary teams may offer more than the simple transfer of services, and the approach includes several possibilities [57]:

- secondary care service substitution
- secondary care service addition
- meeting unmet needs – an alternative and often overlooked possibility that the service may meet the needs of patients that were not met previously.

### Why primary care based multidisciplinary teams?

Some studies suggest that the most effective interventions were delivered at least partially at home [58] and the methods used by secondary care based teams are fully applicable for primary care services, for example, monitoring compliance with treatment, enhancing patient self-care, and providing patient education [59, 60].

A heart failure clinic disease management model was adapted for use in the primary care setting in Oregon (USA) [61]. A heart failure clinic staffed by 2 internists and their nurses was established in a large primary care practice. Medical care and pharmacotherapy were based on national guidelines. Primary outcomes included quality of life, functional class, and all-cause hospital and emergency room admissions 12 months before compared with 12 months after enrolment; a secondary endpoint was patient satisfaction. The results showed reduction of emergency room visits or all-cause hospitalizations, improvements of quality of life and high patient satisfaction. This heart failure disease management model, designed for patients and providers in an primary care setting, was feasible and successful. Another recent intervention in the US compared home-based management of chronic heart failure with clinic-based management [62]. Home based intervention was found not superior to clinic based care in reducing all-cause death or hospitalization. However, Home based care was associated with significantly lower healthcare costs, attributable to fewer days of hospitalization.

Primary care based models have several advantages. Most of older patients with chronic heart failure have other co-morbidities and psychological issues whose monitoring and management is usually best provided by GPs [42]. Three elements important for effective management in primary care settings irrespective of the particular model, appear to be; 1. trained specialist nurses, 2. education of patients and caregivers about chronic heart failure, and 3. ready access to clinicians trained in chronic heart failure [58]. Primary Care physicians are positioned best for easy accessibility and ensuring the continuity of care.

### Controversial evidence

A recent Cochrane review of randomised controlled trials shows that amongst patients with chronic heart failure who have previously been admitted to hospital for this condition, there is now good evidence that case management type interventions led by a heart failure specialist nurse reduces heart failure related readmissions after 12 months follow up, all cause readmissions and all cause mortality [63]. However, the review states that it is not possible to say what the optimal components of these case management type interventions are, although telephone follow up by the nurse specialist was a common component.

A number of studies showed that multidisciplinary strategies for the management of heart failure patients are cost-saving, reduce mortality, heart failure related hospitalisations and all-cause hospitalisations [58, 59] as well as improve the functional status of patients [60].

A review of 29 randomised trials of multidisciplinary management strategies for patients with heart failure [59] reported that these programs were associated with 27% reduction in heart failure hospitalisation rates and 43% reduction in all reason hospitalisation rates for patients with Chronic Heart Failure. Those strategies that incorporate specialised follow-up by a multidisciplinary team or in a multidisciplinary heart failure clinic also reduce all-cause mortality by about one-quarter.

In contrast, another randomized controlled trial looked into outcomes of an intervention that included discharge planning, a primary care physician hospital visit, and assistance with follow-up appointment scheduling before hospital discharge [64]. This was associated with increased rather an decreased hospitalizations and hospital days compared with a control group.

In a real-world population-based study in Canada, which included more than 14 thousand patients with chronic heart failure, it was found that multidisciplinary heart failure clinics were associated with a decrease in mortality, but an increase in readmissions [65].

Similar results are demonstrated by a review of randomised controlled trials, which was undertaken to determine whether the management of heart failure by specialized multidisciplinary heart failure disease-management programs was associated with improved outcomes [66]. Of the 11 studies selected, nine involved specialized follow-up using multidisciplinary teams and the remaining two involved follow-up by primary care physicians and telephone. These studies involved 1,937 heart failure patients with a mean age of 74. The follow-up period ranged from no follow-up (one study) to 1 year (one study), Seven of the nine studies did not show any significant association between intervention and reduced hospitalization, but the two studies that used follow up by primary care physicians and telephone failed to show any significant reduction. In fact, one of the studies demonstrated a higher risk of hospitalization for patients receiving intervention.

The reason of the controversy in the evidence above might be attributed to the differences in definitions of the management of patients with Chronic Heart failure [67]. Although there seems to be a strong common understanding regarding the role of multidisciplinary teams, as per National guidelines used in UK, Australia and US [68–70], the detailed components of these interventions and their impact on the care of patients with chronic heart failure are far less clear. Some evidence is available on the efficiency of certain components of multidisciplinary interventions, indicating that enhanced self-care, follow-up monitoring by specially trained staff and access to specialised heart failure clinics appear to be most efficacious approaches [59].

### Risks and considerations

The existing evidence, although supporting the possibility of provision of services for people with CHF in Primary care, does not provide sufficient evidence on effect of certain components of multidisciplinary interventions on health care services use or patient outcomes. It is important to agree that not *all* MDT interventions may be efficient. The interventions should be based on existing guidelines, with clearly defined structure, actions and responsibilities, as well as monitoring of the effects and impact on patient care and health outcomes.

Other risks relate to the fact that the GPs are often operating in an isolated environment with great financial pressures to restrict referral to hospital. The relationship between the GPwSI and the local cardiologists is pivotal in allowing opportunities for informal exchange of information and case discussion [53].

A particular concern is the extent to which increases in primary care workload are being supported by shifts of resources into primary care [71]. This is likely to influence the willingness of the primary care sector to support the overall policy goal of a shift to primary care [72]. Poorly targeted resource allocation has implications for equity and efficiency, and is considered one of the reasons why increased public spending for health has not proportionately improved equity of access and outcomes, and has had less impact on health status than expected [3].

The organisation of additional services within already busy GP practices should avoid deleterious impact on existing services. In view of the high workload of health care professionals, additional staff, equipment, protected learning time, protected time for meetings with consultants should be considered and financed accordingly. Improving technical opportunities for communication between primary care and secondary care specialists such as telehealth, email and others are potential supports for the system, increasing patient safety and hopefully health outcomes. It has been already shown that telemonitoring and structured telephone support appear to lead to benefits for patients with chronic heart failure [73].

As a significant part of the approach, patient preferences need to be weighed against the clinical and resource arguments surrounding changes, in where and how care is delivered. It will also require mechanisms to look into patients’ needs and wishes and to monitor quality of services for patients after any shift has occurred [30].

## Summary

Current research supports the idea of the management of certain chronic health conditions in primary care based on the integration of GPs and specialists into multidisciplinary teams, availability of reliable evidence about cost-effectiveness, health care outcomes, patient preference and incentives for GPs. Evaluation of such schemes is mandatory, however, to ensure that the expected benefits do materialise.
